# Post-Activation Performance Enhancement and Motor Imagery Are Efficient to Emphasize the Effects of a Standardized Warm-Up on Sprint-Running Performances

**DOI:** 10.3390/sports11050108

**Published:** 2023-05-22

**Authors:** Valentin Rumeau, Sidney Grospretre, Nicolas Babault

**Affiliations:** 1 INSERM UMR1093-CAPS, Université de Bourgogne, UFR des Sciences du Sport, F-21000 Dijon, France; valentin.rumeau@orange.fr; 2 EA4660-C3S, Université de Franche-Comté, UFR des Sciences du Sport, F-25000 Besançon, France; sidney.grospretre@univ-fcomte.fr; 3 Centre d’Expertise de la Performance, Université de Bourgogne, UFR des Sciences du Sport, F-21000 Dijon, France

**Keywords:** agility, repeated sprint ability, reaction time, subjective fatigue, ice hockey

## Abstract

Warm-up routines include various tasks focused on the peripheral contractile properties and nervous motor command. This present study was aimed at investigating the acute effects of different warm-up routines, emphasizing either peripheral (post-activation performance enhancement, PAPE) or central (motor imagery, MI) contributions on sport-specific tasks. Eleven young female athletes took part in this cross-over, randomized, controlled trial. They underwent three experimental sessions composed of a standardized warm-up followed by 10 min of (1) rest (CONTROL), (2) maximal concentric leg press (PAPE), or (3) mental repetitions of sprint tasks (MI). Post-tests consisted of reaction time, arrowhead agility test, 20 m sprint, repeated sprint ability, and NASA-TLX fatigue questionnaire. PAPE and MI significantly enhanced the arrowhead agility test (*p* < 0.001 and *p* = 0.012, respectively) and repeated sprint ability (*p* = 0.002 and *p* = 0.035, respectively) compared to CONTROL, without any difference between PAPE and MI. The 20 m sprint time was better after PAPE as compared to MI (*p* = 0.005) and CONTROL (*p* < 0.001), without any difference between MI and CONTROL. Reaction time and the NASA-TLX questionnaire were not affected by the warm-up modalities (*p* > 0.05). PAPE was the most efficient to optimize warm-up due to its greater peripheral contribution that would improve muscle contractility. MI specifically improved the imagined tasks mostly by central contribution.

## 1. Introduction

Proposed before training or competitions, warm-up is an unavoidable primary step to prepare athletes for subsequent exercise, to improve their performance, and decrease the risk of injury [1]. Warm-up mechanisms are either thermo-dependent or independent of temperature [2]. They generally include alterations in the cardiovascular and neuromuscular systems as well as cognitive changes [3,4] that will impact the subsequent physical, cognitive, and technical performance. Warm-up can be either active or passive depending on the presence or absence of any physical activities, respectively [2].

The traditional warm-up strategies are mainly active and usually include various activities at low or high intensities such as low-intensity running or cycling followed by some flexibility and activity-specific exercises [5]. Amongst the possible activities, numerous studies recommended using very high-intensity contractions at the very end of the warm-up routines to potentiate the muscular system. These short conditioning contractions include maximal loads [6], submaximal loads [7], or even body-weight exercises especially when the aim is to optimize the maximal contraction velocity [8]. Various exercises have been proposed such as resistance training exercises (e.g., leg press or snatch) [9], jumps, or sprints [10]. Frequently named post-activation potentiation, such high-intensity exercises are now called post-activation performance enhancement (PAPE) without any direct evidence of the specific post-activation potentiation effect [11]. It recently became an increasingly popular and time-efficient method to acutely enhance the peripheral muscular system including power production or even explosivity [12]. However, the effects of PAPE on activity-specific tasks remain debated [13].

In contrast, passive warm-up does not require any voluntary contractions. It is achieved, for example, by using clothes or hot environments, and has shown great effectiveness for neuromuscular function optimization [2]. Amongst the various passive warm-up modalities, motor imagery (MI) could be of interest for its central effect. MI uses all the senses to create or recreate an experience or overt action in the mind without any concomitant body movement [14]. This technique has become one of the most widely used simulation tools and performance enhancement strategies among sports psychological interventions [15]. MI-based warm-ups have numerous effects, for instance, by increasing motivation and decreasing anxiety, improving mental skill use, motor learning, and motor performance, for example, while increasing force and coordination [14,16]. From a neuromuscular point of view, it is now admitted that MI acutely increases the excitability in a large part of the motor system, i.e., of motor brain regions and of some circuits in the spinal cord that are normally activated during real movements [17,18]. However, whether MI could substitute some activity-specific exercises and optimize warm-up routines, while saving potential energy output and consequently limiting fatigue, remains to be investigated.

PAPE and MI easily appear diametrically opposed in terms of their peripheral or central contributions, respectively. Nevertheless, these two modalities seem to be good alternatives for optimizing warm-up routines and subsequent muscle performance. Due to their characteristics (very brief high-intensity contractions for PAPE or no contraction with MI), PAPE and MI could both increase neuromuscular performance while reducing warm-up time and reducing potential muscle fatigue consequent to the repetitions of muscle contractions [19]. However, the acute effects of these two strategies implemented within a comprehensive warm-up on activity-specific tasks remain to be clarified. Therefore, the aim of this present study was to investigate the acute efficiency of different comprehensive warm-up routines, emphasizing peripheral (PAPE) or central (MI) contribution to some sport-specific tasks. We, therefore, compared a traditional warm-up to the same standardized warm-up associated with either PAPE or MI on agility, sprint, repeated sprint ability, reaction time, and subjective fatigue. We hypothesized that PAPE and MI warm-ups would be more efficient than a traditional warm-up. Since both PAPE and MI warm-ups are applicable in real-world settings, this study will provide helpful knowledge to practitioners and coaches for warm-up optimization.

## 2. Materials and Methods

### 2.1. Participants

Eleven women from the French U20 national ice hockey team were included in this study. Participants’ characteristics are presented in Table 1. During the total duration of the study, the physical load was standardized for all participants. None reported injuries within the last three months or any clinical contraindication that could affect data collection or experimental results. Prior to participation, they were fully informed about the purpose of the study and the experimental procedure. However, participants were blind to our *a priori* hypothesis. All signed an informed consent form (signed by their parents for those athletes under 18 years old). This study was conducted according to the declaration of Helsinki. Approval was obtained from the CERSTAPS (ethics committee for research in sports sciences; IRB00012476-2022-15-03-165). The sample size was calculated a priori using G*Power (version 3.1.9.6, free software available at https://www.psychologie.hhu.de/arbeitsgruppen/allgemeine-psychologie-und-arbeitspsychologie/gpower.html, accessed on 10 October 2019) with the following parameters: effect size of 0.15 (partial-eta-squared, ηp2), power of 0.8, probability error of 0.05. A sample size of 11 individuals was indicated.

### 2.2. Experimental Design

**Figure 1:**
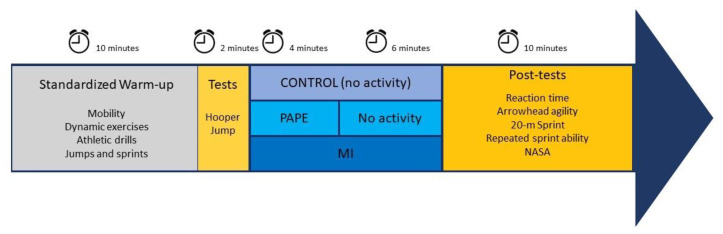
Overview of the study design. CONTROL: control condition only including the standardized warm-up. PAPE: post-activation performance enhancement condition. MI: motor imagery condition.

This study was a cross-over, randomized (www.randomizer.org, accessed on 2 April 2021), controlled trial. All participants attended the French ice hockey training center on four separate occasions (familiarization and three experimental conditions) with a minimum of 24 h in between. During the three randomized experimental sessions, the participants completed a 10 min standardized warm-up. After 1 min of recovery, participants performed two countermovement jumps that served as a control of the baseline fitness state. Then, the participants completed one of three experimental conditions according to randomization: no activity (CONTROL), post-activation performance enhancement (PAPE), or motor imagery (MI) (Figure 1). Immediately after these experimental conditions, participants took part in post-tests. The tests included an arrowhead agility test, a 20 m sprint, a repeated sprint ability test (RSA), a simple reaction time task, and a NASA task load index questionary (NASA-TLX).

### 2.3. Procedures

All participants attended the French ice hockey training center on four separate occasions (familiarization and three experimental conditions) with a minimum of 24 h in between. All experimental sessions were performed at the same hour of the day (in the morning, 2 h after a standardized breakfast).

The familiarization session aimed to (i) explain the experimental procedure; (ii) determine anthropometrics (age, height, body mass, and percentage of fat mass), leg press 1-RM (repetition maximum), and MI ability; and (iii) familiarize participants with the different tests and warm-up exercises. Body mass and percentage of fat mass were measured using a Tanita BC420 (Tanita, Tokyo, Japan). During this session, participants completed the 3rd version of the Movement Imagery Questionnaire (MIQ-3) [20] to determine volunteers’ self-estimation of MI ability. The initial mean MIQ-R score was 13.6 ± 1.8 out of 21 indicating a good imagery capacity in all participants.

Control condition (CONTROL): This session consisted of a 10 min standardized warm-up followed by 10 min of rest with participants in a sitting position. During the standardized warm-up, participants completed two minutes of joint mobility (shoulder rotations, hip exterior/interior rotations, hip flexion/extension, hip abduction/adduction, pelvis rotations, knee rotations, and ankle rotations). Once finished, participants completed three minutes of dynamic exercises including three series of 20 lunges interspersed with 30 s of front squats, three series of 10 squats interspersed with 12 repetitions of bungee pulls at the chest (starting position, arms parallel to each other and to the floor). Then, participants completed three minutes of athletic drills (2 × 28 m for each exercise) including heel-to-toe, tipping, and bouncing strides. Finally, participants completed two minutes of high-intensity exercises including three jumping squats and three sets of 28 m of running at 75%, 85%, and 95% of the maximal sprinting velocity with 30 s between each exercise. Immediately after the standardized warm-up, participants remained seated for 10 min.

Post-activation performance enhancement (PAPE): After the standardized warm-up (as described above), participants were asked to perform maximal contractions on the leg press machine (Pure Leg Press, Technogym, Technogym France Sas, Issy les Moulineaux, France). Participants were first asked to complete a 15 s isometric contraction with a 120° joint angle at 85% of their subjective maximum. After 1 min of recovery, participants then performed five concentric contractions at 90% of their 1-RM. Finally, participants were seated for the remaining 6 min.

Motor imagery (MI): After the standardized warm-up (as described above) participants were asked to perform MI under the supervision of the same experimenter. The MI condition consisted of mentally completing, in succession, two arrowhead agility tests (one left-side trial and one right-side trial) followed by one RSA test. Each mental trial was interspaced by 30 s of recovery. Participants were instructed to imagine executing their maximal performances. Each imagined trial was preceded by an oral signal that was given by the experimenter. Participants were asked to indicate when they finished imagining the movement. 

#### 2.3.1. Post-Standardized Warm-Up Measurements

One minute after the standardized warm-up, participants needed to complete a subjective Hooper-Mackinnnon questionnaire and needed to perform maximal vertical jump tests. This served as baseline values to control for the fitness level and to ensure a day-by-day reproducibility. The Hooper-Mackinnnon questionnaire was composed of eight items (fatigue, psychological stress, sleep, muscle pain, enthusiasm for training, irritability, health, and recovery), each scoring from 1 to 7 [21]. The sum of each individual score was calculated and served as the Hooper index during statistics. The vertical jump test consisted of two counter movement jumps (90° knee joint angle and arms akimbo) interspersed with 1 min of passive recovery. The jump height was measured in cm with optometric cells placed on the ground (Optojump Next, Microgate Italy, Bolzano, Italy). The best trial was retained for analyses.

#### 2.3.2. Post-Intervention Tests

Immediately after the completion of the three experimental conditions, participants conducted post-tests in the same order. Tests included a simple reaction time task, an arrowhead agility test, an RSA test, and a 20 m sprint test. One-minute rest was permitted between these tests. Then, participants fulfilled the NASA-TLX questionnaire.

Simple reaction time task: The participants stood with one foot placed between two optometric cells (Optojump Next, Microgate Italy, Bolzano, Italy). The participants needed to remove their feet from the floor as soon as the Optojump Next application displayed a red light on the computer. The time between the appearance of the red light and the absence of foot contact was measured. Each participant repeated two sets of three trials with 45 s rest between sets. 

Arrowhead agility test: This test consisted of (1) running 10 m in a straight line to the first training cone; (2) passing outside the training cone and turning 90° to the right or left side (predetermined depending on the randomization) to a second training cone located at 5 m; (3) by the outside of this second training cone, a change in the direction of the race to proceed to a third training cone located at 15 m from the start; and (4) finally, sprinting to the starting line. Participants had to perform this test once, as fast as possible. One-minute rest was permitted between the left- and right-side trial. The order was always the same for a given individual. The time was measured using one pair of photoelectric cells (Brower timing system, Draper, UT, USA). Participants started the test in a standardized position (forefoot of the driving side on a white line placed 5 cm below the starting line).

The 20 m sprint and repeated sprint ability (RSA) tests: Each participant repeated six straight sprints over 20 m, with 20 s of passive recovery between sprints. The duration of each sprint was measured using two pairs of photoelectric cells (Brower timing system, Draper, UT, USA). Participants started each sprint in a standardized position (forefoot of the driving side on a white line placed 5 cm below the starting line). The total time for the six sprints was considered for analyses of the RSA. The first sprint was used to measure the maximum 20 m speed.

NASA-TLX Questionnaire: The NASA-TLX questionnaire is a subjective questionnaire that assesses internal workload with six items (mental demand, physical demand, temporal demand, performance, effort, and frustration). Increments of high, medium, and low estimates for each item result in 21 gradations. Participants were asked to answer questions by ticking feeling boxes. The points were then added, and the total was retained. 

### 2.4. Statistical Analyses

The normality and sphericity of the data were tested and confirmed by the Shapiro–Wilk and Mauchly’s tests. All variables (Hooper index, CMJ, reaction time, NASA-TLX index, arrowhead agility test, 20 m sprint time, and RSA performance) were analyzed using a one-way repeated-measures analysis of variance (ANOVA) with warm-up as the main factor (CONTROL vs. PAPE vs. MI). In case of a significant effect, a post hoc test with Bonferroni correction was performed. In addition, effect sizes were quantified. Partial-eta-squared (η_p_^2^) was calculated from ANOVA results, with values of 0.01, 0.06, and above 0.14 representing small, medium, and large differences, respectively [22]. Subsequently, qualitative descriptors of standardized effects were used for pairwise comparisons with Cohen’s d <0.5, 0.5–1.2, and 1.2 representing small, medium, and large magnitudes of change, respectively [22]. *p* < 0.05 was taken as the level of statistical significance for all comparisons. Absolute values are expressed as mean ± SD or mean difference with 95% confidence intervals (95%CI). Statistics were performed using the JASP Software (version 0.13, JASP Team (2020), University of Amsterdam). 

## 3. Results

Baseline values for the CMJ and Hooper questionnaires did not show any significant differences between conditions. CMJ height for CONTROL, PAPE, and MI were 28.3 ± 2.4 cm, 28.5 ± 2.3 cm, and 28.8 ± 3.3 cm, respectively (*p* = 0.226, η_p_^2^ = 0.143, small). Hooper values for CONTROL, PAPE, and MI were 24.2 ± 6.5, 24.0 ± 6.2, and 25.7 ± 5.6, respectively (*p* = 0.464, η_p_^2^ = 0.074, small). For post-tests, the one-way ANOVA did not show any significant differences for the reaction time (*p* = 0.185, η_p_^2^ = 0.075, small) nor for the NASA-TLX questionnaire (*p* = 0.314, η_p_^2^ = 0.109, small). In contrast, significant warm-up effects were observed for the arrowhead agility test left side (*p* = 0.002, η_p_^2^ = 0.579, medium), arrowhead agility test right side (*p* < 0.001, η_p_^2^ = 0.597, medium), 20 m sprint (*p* = 0.002, η_p_^2^ = 0.561, medium), and RSA (*p* = 0.008, η_p_^2^ = 0.454, small).

**Figure 2:**
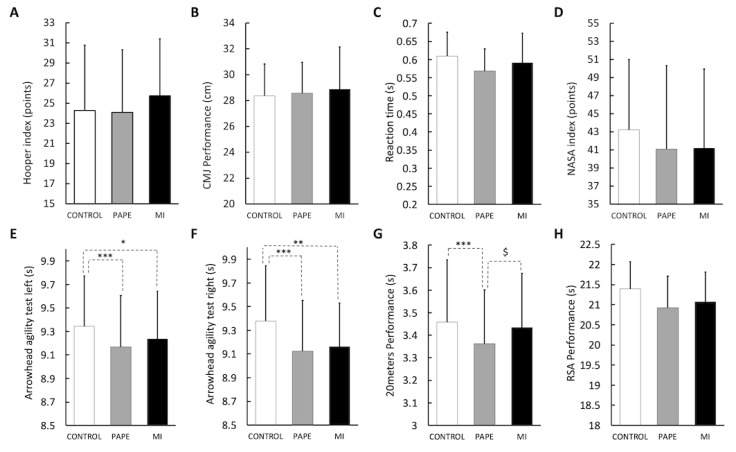
Mean values (±SD) of the Hooper questionnaire (**A**); counter movement jump (CMJ) (**B**); reaction time (**C**); NASA-TLX questionnaire (**D**); arrowhead agility test on the left side (**E**); arrowhead agility test on the right side (**F**); 20 m sprint (**G**); and repeated sprint ability (RSA) test (**H**). CONTROL: control conditions including the standardized warm-up. PAPE: post-activation performance enhancement condition. MI: motor imagery condition. *, ** and ***: significant differences with CONTROL with *p* < 0.05, *p* < 0.01 and *p* < 0.001, respectively. $: significant differences between PAPE and MI with *p* < 0.05.

For the arrowhead agility test, post hoc analyses revealed that time was significantly lower for PAPE and MI as compared to CONTROL (Figure 2). This test, when performed on the left side, revealed that PAPE (mean difference (95%CI): 0.174 (0.086; 0.262), d = 1.583, large, *p* < 0.001) and MI (mean difference (95%CI): 0.110 (0.022; 0.197), d = 0.984, medium, *p* = 0.012) induced significantly lower values than CONTROL. No difference was observed between PAPE and MI (mean difference (95%CI): −0.065 (−0.152; 0.023), d = −0.580, medium, *p* = 0.207). When performed on the right side, PAPE (mean difference (95%CI): 0.255 (0.123; 0.387), d = 1.522, large, *p* < 0.001) and MI (mean difference (95%CI): 0.216 (0.084; 0.348), d = 1.292, large, *p* = 0.001) induced significantly lower values than CONTROL. No difference was observed between PAPE and MI (mean difference (95%CI): −0.039 (−0.171; 0.093), d = −0.231, medium, *p* = 1.000).

During the 20 m sprint, PAPE induced significantly lower time values than CONTROL (mean difference (95%CI): 0.095 (0.044; 0.147), d = 1.469, large, *p* < 0.001) and MI (mean difference (95%CI): −0.070 (−0.122; −0.019), d = −1.085, medium, *p* = 0.005). No difference was observed between MI and CONTROL (mean difference (95%CI): 0.025 (−0.026; 0.076), d = 0.385, medium, *p* = 0.649). During the RSA test, post hoc analyses revealed that time following PAPE (mean difference (95%CI): 0.472 (0.162; 0.782), d = 1.199, medium, *p* = 0.002) and MI (mean difference (95%CI): 0.330 (0.020; 0.640), d = 0.839, medium, *p* = 0.035) were significantly lower than CONTROL. No difference was observed between PAPE and MI (mean difference (95%CI): −0.142 (−0.452; 0.168), d = −0.360, small, *p* = 0.738).

## 4. Discussion

This present study aimed to investigate the acute efficiency of different comprehensive warm-up routines including PAPE or MI on some sport-specific tasks. The main results were as follows: (1) PAPE induced significant improvements in explosive muscular capacities, agility, and repeated sprint capacities; (2) MI was found to be effective in improving agility and repeated sprint skills; (3) no significant difference was observed between PAPE and MI for agility tests; and (4) PAPE or MI were ineffective to change reaction time and subjective fatigue assessment (NASA-TLX) as compared to CONTROL. These results confirmed our a priori hypothesis and demonstrated that PAPE and MI could be used as warm-up modalities to emphasize the effects of a traditional warm-up. 

The first main result of this present study was the positive effects of PAPE in addition to the standardized warm-up on all sprint-running qualities. In this present study, PAPE was achieved using maximal concentric knee extensions. Numerous studies have already observed such beneficial effects to increase maximal muscle strength and overall muscle function [12,23]. For instance, some authors previously observed increased arrowhead agility tests and 30 m sprint performances after submaximal and maximal half-squats [23]. Similarly, half-squats followed by vertical jumps have been shown to increase ice sprint performance [24]. Previous studies also observed increased repeated sprint ability after conditioning activities [23,24]. Body-weight plyometric exercises produced a similar effect [25]. With near-maximal muscle activation, the type of exercise did not seem to significantly impact the subsequent increase in muscle performance as shown following eccentric overload or weightlifting exercises [26]. 

However, and in contrast to the often-suggested post-activation potentiation mechanism, PAPE is a long-lasting phenomenon with potentially positive effects being between 5 and 30 min after the conditioning exercise [6,7,27,28]. However, the larger effects are expected between 5 and 10 min after the conditioning contractions [11]. Authors have often concluded that a recovery period should be programmed to alleviate the likely fatigue originating from the preceding maximal contractions [25,29]. For instance, Gilmore et al. [12] observed increased bat velocity in experienced female softball athletes, 6 min after a high-intensity isometric preconditioning. For that reason, in this present study, PAPE was followed by 6 min of rest to optimize its effect.

As compared to the standardized warm-up, our results can be explained by additional changes in muscle temperature, muscle/cellular water content, and muscle activation [11]. After the warm-up, some neuromuscular alterations have been suggested. For instance, in recent studies, authors have observed increases in electromyographic activity of the considered muscles using complexity-based methods [3,30], which suggest an optimized neuromuscular state for subsequent contractions. The authors also suggested that additional specific exercises could exacerbate the neural adaptations [3,30]. In accordance with this hypothesis, the authors concluded that the conditioning contraction following warm-up would produce additional recruitment facilitation that would improve movement quality [31]. Additional neuromuscular measurements including electromyographic activity should therefore be conducted to confirm such a hypothesis.

The second main finding of this present study was that the standardized effects could be emphasized using MI. MI implies a neural activity without any contractions and, therefore, suggests real movements could acutely be improved only with imagination. The positive effects of MI on the neuromuscular system are well documented [18]. For instance, MI has been shown to increase muscle strength, power, and endurance-type effort when used chronically [15,32,33] and sprint performance when used acutely [34]. However, to the best of our knowledge, this present study is the first to show the positive acute effects of MI as an alternative warm-up strategy. From a neurophysiological perspective, it is known that MI acutely increases the excitability of a large part of the cortico-spinal pathway [18] and that these effects can last at least 10 min after the end of MI practice [17]. MI results in activation of brain motor regions [35], especially if participants have great expertise in sports performance, i.e., if they are high-level athletes [36], as in the present study. Recently, it has been shown that MI can have an acute effect on force performance, mainly by inducing a more efficient cortical drive to motor units and optimizing agonist/antagonist coactivation [37]. However, the activation of the motor neural system remains weaker than during voluntary contraction [38]. All these mechanisms could explain our results and particularly the difference between PAPE and the so-observed MI-specific adaptations.

Indeed, it can be noted that MI has only positive effects on the tasks being mentally imagined. Indeed, only performances in agility repeated sprint tests have been enhanced as compared to the standardized warm-up. This confirmed the specific mechanisms of MI. Indeed, MI primarily increases the neural circuit normally activated during actual movements [17]. Here, the tasks used during MI implied some accelerations/decelerations and some direction changes with a considerable complex motricity. In contrast, with a simpler motricity, MI appeared less efficient than actual movements or peripheral pre-conditioning activity (PAPE). Such a conclusion is witnessed by the lack of increase in 20 m sprint as compared to CONTROL, while PAPE increased such sprinting performance. In addition to the specific effect of MI that did not lead to a transfer to other performances, the lower activation resulting from MI as compared to PAPE could, therefore, appear detrimental. Although lower than PAPE for some performances, MI still appears as an interesting tool for subsequent sport-specific activity. Moreover, MI-based warm-ups could have other additional beneficial effects. Previous studies have shown increased psychological, motor, and cognitive skills such as motivation, for example [14,16].

Performing PAPE or MI was ineffective for reaction time as compared to CONTROL. Such a result is not surprising since such a task requires high attentional and neural activation that is not solicited during PAPE or MI. Moreover, a previous study has demonstrated that MI was ineffective for reaction time performance, even if the imagined task was specifically designed for it, i.e., involved to respond as fast as possible to a light stimulus [39]. Finally, PAPE and MI showed similar results as CONTROL regarding the NASA-TLX score. This result revealed that the subjective overall workload was similar between the three experimental conditions and, therefore, confirm that PAPE and MI could be performed without any adverse effects. 

Several limitations can be raised for this present protocol. Apart from the sample size and the lack of neuromuscular physiological measurements, several choices of the warm-up protocol itself could be criticized. For instance, several types of motor imagery exist (visual or kinaesthetic) which could interfere differently with the subsequent motor performance, especially for reaction time [40]. Similarly, numerous protocols for PAPE could be proposed. The choice made in this present study aimed at finding the compromise between the suitability of the conditioning exercise in a warm-up protocol and the theoretically optimal intensity to optimize subsequent motor performance. Nevertheless, as shown by Sanchez et al. [19], PAPE conditioning exercises carried out at 90% of the 1-RM are more effective on subsequent performance than those performed at 60%. 

Diametrically opposed, the peripheral contribution of PAPE and the central contribution of MI both appeared efficient to emphasize the acute effects of a standardized warm-up. PAPE appeared more efficient for the overall muscle function but requires thoughtful programming to avoid any detrimental effects related to likely fatigue produced by the maximal contraction, especially during warm-up. In contrast, the absence of contraction and high neural solicitation related to MI also appeared efficient for tasks with high and precise motricity such as with numerous accelerations/decelerations and direction changes. It must be noted that MI leads to task-specific effects. Therefore, both methods could be implemented during a warm-up routine to optimize its effects and provide superior results than a classic warm-up routine. These two complementary methods present the advantage to be applicable in real-world settings. The combination of both methods could be of interest and requires future experiments. Moreover, the effects of these modalities on injury prevention must be addressed.

## 5. Practical Applications

The results of this study suggested that comprehensive warm-up routines that included MI improved agility and repeated sprinting abilities without increasing subjective fatigue. The warm-up that included PAPE also induced a significant improvement in explosive muscular capacities. Therefore, it is recommended to the practitioner working with competitive athletes whose performance criteria are agility and sprint repetition abilities, to include MI or PAPE in warm-up routines. If explosive muscle capacity is also part of the athlete’s performance criteria, it is recommended to include PAPE in warm-up routines. The addition of MI or PAPE is suggested in these situations as long as it does not increase fatigue levels.

## Figures and Tables

**Table 1:** Characteristics of the subjects.

Characteristics	Mean ± SD (Range)
Age (year)	17.3 ± 1.1 (16; 20)
Height (cm)	163.8 ± 6.6 (149.0; 173.0)
Body mass (kg)	56.6 ± 7.2 (41.9; 69.0)
Percent fat mass (%)	18.1 ± 2.8 (12.6; 22.4)
Weekly training volume (hours per week) - weekly strength training volume (hours per week) - weekly ice hockey training volume (hours per week)	12.9 ± 1.0 (11.6; 14.3) 5.1 ± 3.4 (1.2; 9.1) 7.8 ± 3.0 (3.9; 12.2)

## Data Availability

The authors declare that the dataset is available upon request.
